# Camera on Vessel: A Camera-Based System to Measure Change in Water Volume in a Drinking Glass

**DOI:** 10.3390/s150923847

**Published:** 2015-09-18

**Authors:** Idowu Ayoola, Wei Chen, Loe Feijs

**Affiliations:** Designed Intelligence, Department of Industrial Design, Technische Universiteit Eindhoven, The Netherlands; E-Mails: w.chen@tue.nl (W.C.); l.m.g.feijs@tue.nl (L.F.)

**Keywords:** fluid level measurement, fluid monitoring, fluid imbalance, camera vision, chronic patients

## Abstract

A major problem related to chronic health is patients’ “compliance” with new lifestyle changes, medical prescriptions, recommendations, or restrictions. Heart-failure and hemodialysis patients are usually placed on fluid restrictions due to their hemodynamic status. A holistic approach to managing fluid imbalance will incorporate the monitoring of salt-water intake, body-fluid retention, and fluid excretion in order to provide effective intervention at an early stage. Such an approach creates a need to develop a smart device that can monitor the drinking activities of the patient. This paper employs an empirical approach to infer the real water level in a conically shapped glass and the volume difference due to changes in water level. The method uses a low-resolution miniaturized camera to obtain images using an Arduino microcontroller. The images are processed in MATLAB. Conventional segmentation techniques (such as a Sobel filter to obtain a binary image) are applied to extract the level gradient, and an ellipsoidal fitting helps to estimate the size of the cup. The fitting (using least-squares criterion) between derived measurements in pixel and the real measurements shows a low covariance between the estimated measurement and the mean. The correlation between the estimated results to ground truth produced a variation of 3% from the mean.

## 1. Introduction

The comorbidities associated with the circulatory system of HF and hemodialysis (HD) patients make them prone to fluid imbalance [1,2,3]. In the case of HD patients, fluids are restricted to 1L per 24 h to minimize fluid accumulation between two consecutive dialysis sessions. The amount of fluid accumulated can be estimated by taking the patient’s weight before dialysis and subtracting the weight at the end of the previous dialysis session; this is also called inter-dialysis weight gain (IDWG). High IDWG can lead to symptoms of fluid overload, such as dyspnea, pulmonary edema, decreased appetite, pain or discomfort. High IDWG is also associated with lower overall survival in this patient group. Multiple factors influence the adherence to fluid restriction, such as thirst, personal habits, and social factors. Only 30%–60% of the HD patients are able to adhere to their fluid restriction.

Nonadherence to dietary and fluid restrictions is a common problem in HD or HF patients (amongst others) and is associated with increased morbidity and mortality. Research on nonadherence is associated with inconsistencies in definitions and invalid measurement methods, such as self-reporting. Further research is needed to validate measurement methods and to establish clinically relevant operational definitions of nonadherence [4]. A literature review revealed that more research is needed into the effect of psychosocial, behavioral, and physiological factors that affect adherence [5]. A review in [6] shows the effects of thirst, lifestyle symptoms, and effective interventions in terms of relieving troublesome symptoms in patients. Social factors related to drinking behavior are also difficult to capture. We hypothesize an approach to improve adherence by enriching drinking behavior with information on body fluid retention and fluid excretion. Body fluids can be measured using either body weight or body composition measurement (BCM). While it is challenging to measure fluid excretion in HF patients, in HD patients, fluid is removed during dialysis and can therefore be monitored. Measuring fluid intake remains a challenge due to the diverse range of scenarios of dietary and fluid intake. There are new sensorized devices emerging with the purpose to track fluid intake. *Obli* [7] is designed to track water intake from a stationary bottle. *My Vessyl* [8] is another device, which was designed for people who like to keep their vessel close by. To make it possible for a user to monitor his or her drinking behavior using a portable device while allowing for diverse applications—with a preferred cup, for home and ambulatory use—we conceptualize a small instrument that can be clipped onto the glass. We use a camera clipped to the glass to estimate the amount of liquid. With the monitoring technology, we hope to capture the user’s micro behavioural pattern to intervene in an integrated and smart way. This can hopefully help to inform the user, reduce thirst, and improve adherence.

In this paper, we present an empirical approach that involves using a camera to infer the real level of water and the change in volume in a glass, possibly due to drinking. Firstly, we present the state-of-the-art devices, then we describe the general approach and assumptions, followed by a feasibility study. Finally, the method is described and the inferred results are presented.

## 2. State-of-the-Art

**Figure 1:**
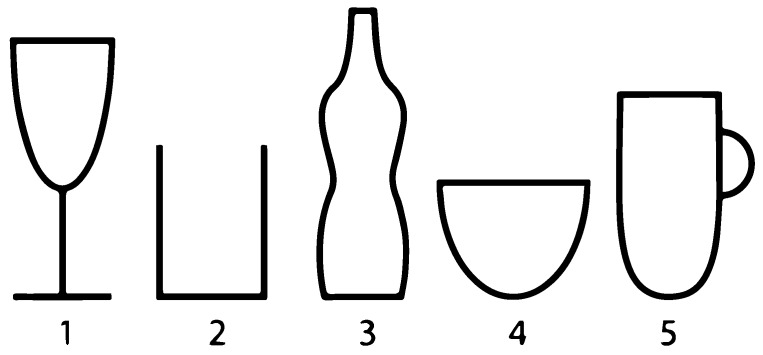
Shapes of drinking vessels.

We present a survey that identifies the obvious sensor technologies that are applicable to estimate the liquid volume change in a container or vessel used for drinking. We employ three criteria to describe and compare the suitability of the method from technology and user perspectives. These criteria include:

- Technology Application and Accuracy—refers to how the sensor is relevant or applicable to different vessels or shapes in estimating the change in the liquid volume. Examples of the shapes are shown in Figure 1.
- Technological feasibility—refers to the technological challenges, and the integration possibilities of the sensor device.
- Human factor—refers to the factors affecting the use of the device from the users’ perspective. These factors may include, ease of transportation, the ease to attach onto the vessel, cleanability, and the ability to use for ambulatory or stationary situations.

The sensor technologies mentioned on Table 1 can be adopted for different scenarios according to the user’s needs. For example, the weighing method is more suitable for home use wherein the user has more control over the movement and positioning of the cup. The clip-on methods—using CMOS camera or the proximity sensor—can also be useful for home use if it can be attached in a simple and fast way. The band or clip-on methods seems reasonable for outdoor usage (*i.e.*, at stand-up parties, drink at the bar, *etc.*). The band and clip-on devices may allow the user to move freely with the cup and to continually track the liquid volume while holding and refilling the cup. The flow method may be more suitable for sport or dynamic situations (*i.e.*, cycling) since it allows the user to drink directly from the bottle.

Fluid consumption for HF or HD patients mostly occurs at home and occasionally at social events. HD patients undergo dialysis 2–4 times per week. Each dialysis session may last for up to 4 h. During dialysis, drinking also occur that should be included in the monitoring. The clip-on methods have potential to be portable for easy transportation to be occasionally useful for out of home use. It will allow the user to use his or her preferred drinking cup and can be applied in stationary or ambulatory situations. This makes the clip-on method a good modality for monitoring drinking for these patients. In this paper, we focus on using a low-resolution camera that can be clipped unto a conically shaped glass to continuously monitor the changes in the liquid volume.

**Table 1:** An overview of sensor technologies applicable to monitor water/fluid intake.

Sensor Technology/Method	Example Product/Image	Technology Application and Accuracy	Technological Feasibility	Human Factor
**Immersion Method** Liquid pressure transducer: Measures the force required to stop the fluid from expanding.	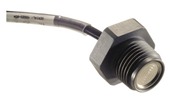	The sensor can measure depth accurately in tanks and basins. It is not suitable in small vessels (as shown in Figure 1) due to its bulkiness.	The technology is well applied in the industry. They are not so miniaturized, therefore, difficult to integrate in the vessels; nevertheless, integration within the vessel might be required.	The sensor requires contact with the liquid to measure. This can make it difficult to clean. The sensor may be required to be integrated in the vessel which makes it bulky to transport. When integrated, it can be suitable for stationary and ambulatory use.
**Clip-on Method** Proximity sensor (*i.e.*, ultrasonic distance sensor): Measures the distance from the surface of the liquid to the top of the container.	Trago [9]: Is a design that incorporates a proximity sensor within a bottle cover. It detects the distance from the bottle cover to the surface of the contained liquid. The prototype is in development phase on Kick-starter.	The sensor can be made to fit on all forms shown in Figure 1. However, the shape of the vessel should be known and calibrated accordingly to assure accuracy of the measurements. Better accuracy is achieved when the container has a regular form.	The sensor is required to be miniaturized to be integrated into the vessel cover or as a clip-on device. The perpendicular alignment of the sensor is also very important to avoid diagonal readings, which makes it challenging.	It can be contactlessly used as a clip-on or a bottle cover that can be easy to attach. The user will need to specify the shape of the vessel for proper calibration. It will be easy to clean as a separate device. When designed as a clip-on device, it should not be in the way of drinking and should not be contaminating. The device can be made portable as it depends on the design.
	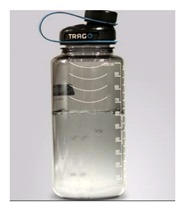			
**Clip-on Method** CMOS Camera: The camera is used to capture images within the vessel to approximate the liquid surface area and the change in the liquid level.	Camera on Vessel: The design incorporates a camera that can be clipped on the edge of a vessel type 1, 2, 4, 5 as shown in Figure 1. (This paper present the development of this device type)	Calibrated to measure the change in liquid volume for a single cup.	It requires image processing and geometric conversions. The image quality is also dependent on the lighting conditions. Miniaturization is desired.	When miniaturized, it can be used without obstructing drinking. When miniaturized, it can easily be transported. It is portable for outdoor use. It provides the possibility to identify the content of the glass (*i.e.*, water, milk, *etc.*).
	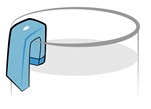			
**Band Method** Capacitive level sensor: Capacitive level sensors consist of two plates. The capacitive value between the plates will change depending on the liquid level in the vessel. The change in value also depends on the dielectric property of the liquid.	Capacitive Sleeve: With this design, the capacitor plates are integrated into a sleeve-like design. It measures the capacitive liquid level from the outside walls of the container. Device is in research phase.	The sleeve can easily be wrapped around a regularly shaped/ elongated vessels such as 1–3, 5 in Figure 1. Capacitive level sensing is accurate depending on the area of the plates and the dielectric material. Water has a high dielectric constant that is highly sensitive to the change in liquid level. While, coffee has a low dielectric constant that is less sensitive.	Capacitive level sensing is well known and applied in the industry that can be applied in the design of the capacitive plate. A major challenge is to shield the capacitive field from external capacitance such as touch. This will allow proper measurements. The technology can only measure the liquid level and not the volume. It should be used for known diameters only. Otherwise, an additional sensor should be used to measure the diameter of the vessel. The electronic should be flexible and miniaturized for proper integration in the sleeve.	The user is required to wrap the sleeve around the vessel before drinking. The device supports good hygiene, as it is not required to have contact with the liquid or the drinking area. It can be applied to a variety of containers suitable for various scenarios or applications. The form factor makes it possible to use on the go. It can be obtrusive if the sleeve is not properly designed/ integrated.
	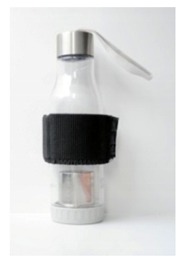			
**Band Method** Accelerometer/ gyroscope: Measures movement and orientation due to gravity.	THE + HUG [10]: A sensor band that tracks water intake by movement. 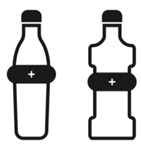	May apply to all shapes. It can only measure drinking action and cannot measure the quantity that was drunk.	Simple and accessible sensor. It is challenging to estimate drinking based on movement.	It is non-obtrusive. It would not bother the user while drinking.
**Base Method** Strain gauge/air pressure sensor: Measures the change in weight of the vessel.	Obli [7]: A device available on the market used for tracking drinking. It measures the weight of a fixed bottle and assumes the weight of water. It can give notifications when the user does not drink. 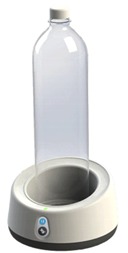	The weighing technique can be used for different vessels when placed on flat surface. The measurement should be re-calibrated when used with different weights. Can perform very accurate measurement.	Weight sensors are accessible and industrially well known. It is easy to calibrate.	Requires discipline, only works if vessel is placed on the sensor each time before and after drinking. It is most applicable for home use. Not obstructive, does not bother user while drinking Simple and easy to apply.
**Tubular Method** Flow sensor: Measures flow from a drinking tube.	BluFit [11]: The sensor is placed on the cover of the bottle. It measures the amount of liquid that flow through the tube as the user drinks. Device is in development phase. 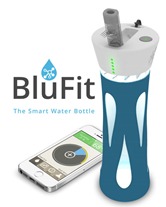	The sensor is limited to a single bottle. It can only be used on a bottle or a container that has a lead. Flow sensors are generally accurate.	Flow sensors/techniques are accessible and industrially well known.	It measures when the user actually drank from the bottle. Requires user to drink all liquids from the same bottle.
**Manual Method** Drink counting App: The user can manually log his drinking activities using a mobile-phone application.	Waterlogged [12]: With the mobile application, the user can manually log his drinking activities. 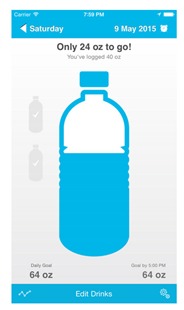	It does not matter what kind of vessel used for drinking.	Many applications are available and do not constitute a major technical challenge. It is difficult to capture drinking at the moment it occurs.	It is unreliable for the user to estimate the size of every drinking glass and to register appropriately. It is difficult for the user to keep track of multiple drinking activities. It requires a subjective estimation.

## 3. Approach

A camera is placed in such a way that it captures the inside of a glass, in order to estimate the difference in volume due to changes in liquid level as the user drinks. We manually measure the liquid level and diameter of the glass for each level, in millimeters, as the ground truth. We also measure the liquid level and the sectional area of the glass for each level, in pixels. We can only dynamically obtain the measurements in pixels as the derived measurement. A correlation metric is then applied to infer the measurements in millimeters. Using the inferred parameters, we are able to estimate the volume difference of the empty portion of the glass in *ml*, which suggests the amount that has been consumed.

### 3.1. Setup and Assumptions

**Figure 2:**
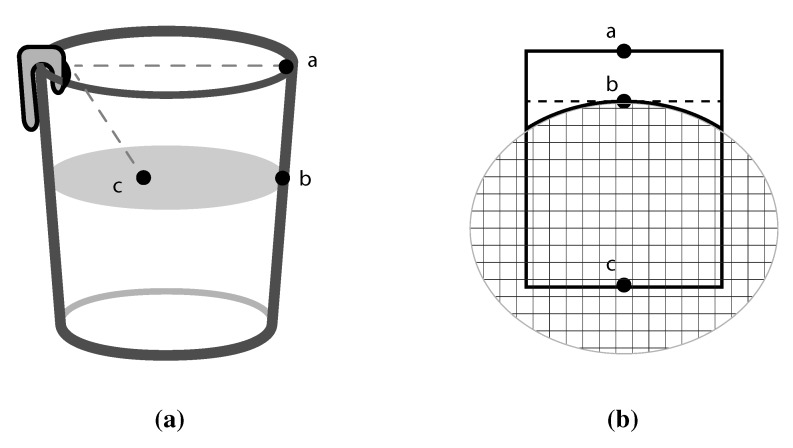
(**a**) Camera setup on the vessel; (**b**) Illustration of the image captured.

We assume a glass that is conically shaped (as most cups are). As illustrated in Figure 2, the camera is affixed on the edge of the glass to capture the inside. As shown in Figure 2b, the image should contain the segment of the arc resulting from the contact of the liquid with the surface of the glass. The resulting view is an arc of an ellipse due to the camera angle. The arc changes its degree of curvature depending on the liquid level and size of the glass. Distance $\bar{ab}$ is the measured level of the liquid in pixels. By using several stages of image processing, the surface of the liquid is reconstructed (assuming an ellipse) using geometric conversions. The etched ellipse in Figure 2 is the ellipsoidal conversion that can be obtained using the present features. The area of the ellipse is the cross-sectional area of the glass in pixels. Thus, we can obtain a volumetric measurement due to the change in liquid volume. When the glass is empty, no level will be present or detectable.

### 3.2. Feasibility Study

**Figure 3:**
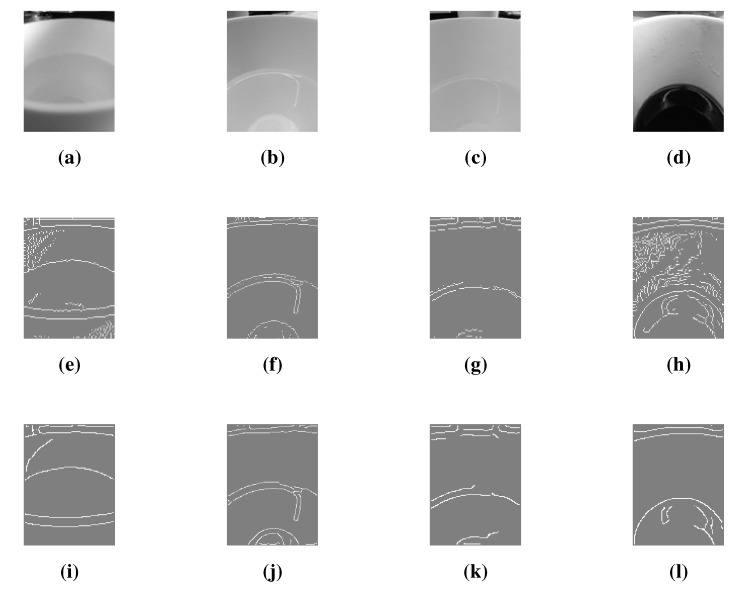
Images with their corresponding edges. (**a**–**d**) Raw images with liquid at different levels; (**e**–**h**) Default Sobel Edge detection, thresh=0.005; (**i**–**l**) Default Canny Edge detection.

We aimed to determine if it is possible to find the water edge in the container under relatively good conditions. We used the back camera of an iPhone 5s to capture the inside of a plastic cup containing water or coffee. This camera was chosen as a quick start and because of its ability to take high quality images. The camera was positioned at an overhead angle to the container, similar to Figure 2. We expected the image to contain edges that depict the liquid level. Multiple images were captured and imported into MATLAB R2013B to perform line-based edge detection using default Canny and Sobel filters. Image capturing and processing are sometimes time, memory and CPU intensive to execute. For this reason, the images were resized to 120×160 and converted to greyscale. A Disk Blur filter was applied to the image using *imfilter* prior to the edge detection to enhance the results. The resultant images shown in Figure 3 were visually analyzed in order to determine whether the desired edges were present. Canny Edge detection showed better results than Sobel Edge detection. However, undesired edges were still present—and sometimes dominant—due to the reflection of the surrounding lights within the container.

## 4. Method

### 4.1. Image Capture

Miniature TTL Serial camera from Adafruits [13] was then selected due to its miniaturized nature and low power consumption. The camera was connected to an Arduino micro-controller [14], which is programmed to control the general functionality of the camera system. The micro-controller streams captured images to a custom NodeJS server [15] running on a 2.5 GHz Intel Core i5 Apple computer (OS X Yosemite). The server then converts the binary images to JPEG format and saves them in an appropriate directory for processing with MATLAB. In the future, the images should be pre-processed or fully processed by the micro-controller before transmitting to an accompanying mobile or desktop application.

### 4.2. Processing

All images were pre-processed, as described in Section 3.2. This section enumerates the steps taken to derive measurements from the images.

#### 4.2.1. Segmentation

**Figure 4:**
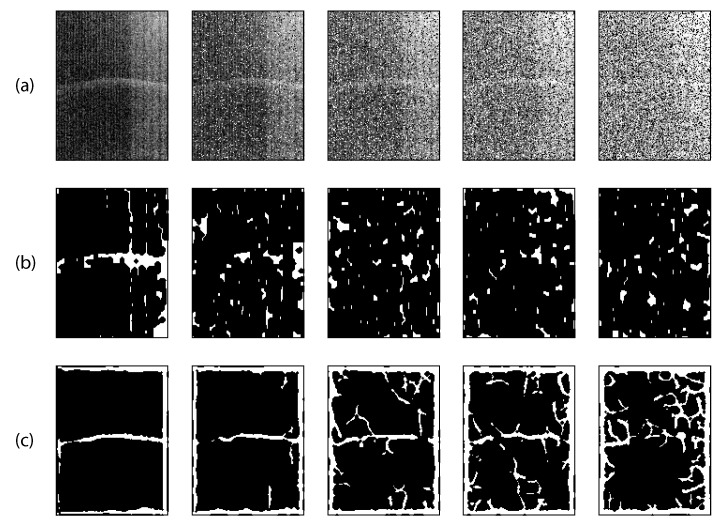
(**a**) Image with *Gaussian* and *Salt and Pepper* added in levels of 0.1; (**b**) Results from *Sobel* filter; (**c**) Results from vesselness filter.

The feature of interest is the tubular structure that denotes the liquid level. The image was pre-blurred using a Disk filter. The Sobel approximation was applied to find the edges because of its high sensitivity, which is needed for the low-resolution image. A morphological filter can produce better results than the Sobel filter and can be tailored to extract the tubular structure. We experimented with multiscale vessel enhancement filtering [16] and implemented the Vesselness function from [17]. The Vesselness function uses the eigenvectors of the Hessian to compute the likeliness of an image region to tubes. We applied Gaussian and Salt & Pepper noise (where the noise density is incremented by 0.1) to systematically worsen the quality of the image (see Figure 4a). The Sobel filter was applied to the noised image as shown in Figure 4b. The Vesselness filter was also applied to the noised image, Figure 4c. The feature of interest was not visible by the second iteration of the Sobel filter. The feature of interest remained visible for all images using the Vesselness filter.

The Sobel filter was used as it was sufficient to detect the feature of interest for most of the images captured. We obtained a binary gradient mask that segments the lines of high contrast in the image. The result was dilated using *imdilate* based on linear structuring elements to close the gaps between connected pixels. The leftover holes due to the dilation process were then filled using *imfill*. Finally, we applied *imerode* to smooth the results and to clean up the segmented elements.

#### 4.2.2. Level Detection

**Figure 5:**
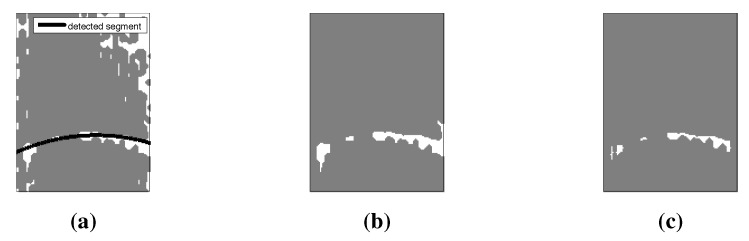
Level detection. (**a**) Gradient mask with detected segments; (**b**) Extracted segments; (**c**) Sorted and smoothed segments.

We expected the pixels that denote the liquid level to be pronounced in the segmentation result. The connected components were obtained using *bwconncomp*. The arc should have a quadratic form of $y=ax^{2}+bx+c$. Given the size of the image, the gradient for a=0.003 and the observed line of symmetry lies within the range -0.58>b<-0.14. *C* is the intersection alongside of the image (size =120×160). To detect the level segment, the segmented image was scanned for every row in the image with the constructed curve. Four steps for *b* at every row were sufficient to detect the connected segments that intersected with the curve (Figure 5a). We selected the scan result with the most pixels and added new points to connect the unconnected components. We iterated through the image again to remove the connected pixels that were too wide on the vertical profile. This helped to reduce false averages during fitting. Finally, the image was smoothened as shown in Figure 5c.

#### 4.2.3. Formulating the Missing Points

**Figure 6:**
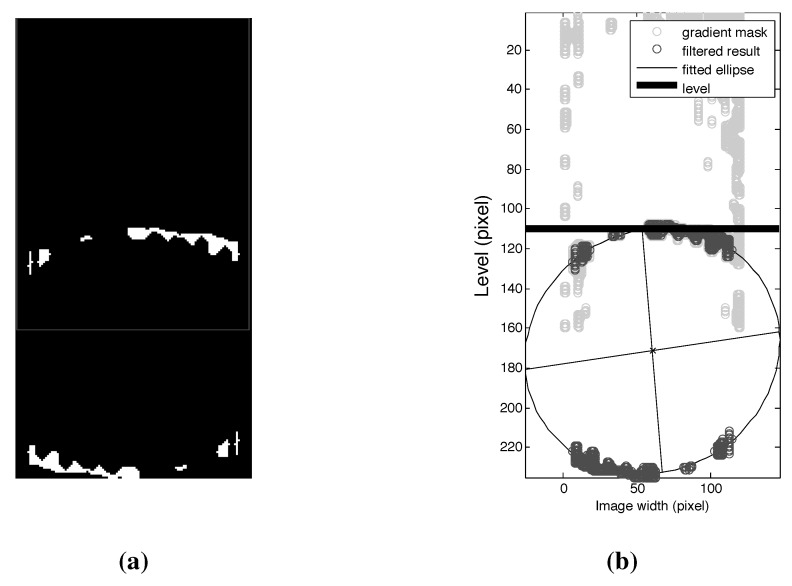
(**a**) Re-constructed image; (**b**) Result of ellipsoidal fitting and level detection.

We assumed that the surface of the liquid was ellipsoidal due to the camera perspective. The extracted segments in Section 4.2.2 is one side of the ellipse; therefore, we rotated a copy to make-up for the opposite side that is not visible in the image, Figure 6a. Using trial and error, a constant value of 50 pixels was chosen as the distance between the rotated segment to the bottom of the image. The trial and error approach was based on the performance of the ellipsoidal fitting and the corresponding measurements in milliliters. The performance increased slightly from a *SE* (standard error of mean) value of 4.26–4.13 mL as the value was changed to 10, 20, … 50 pixels. At 60 pixels, the performance decreased slightly to 4.14 mL.

#### 4.2.4. Ellipsoidal Fitting and Measurements

The surface area of the liquid obtainable in pixels is the area of the ellipse that best fit the extracted segments. *Ellipse Fit* [18] was used to find the best-fit to the ellipse for the given set of points obtained from the previous result in Section 4.2.3. Ellipse Fit uses a least-squares (LS) criterion to find the best fit. The LS estimation is done for the conic representation ($ax^{2}+bxy+cy^{2}+dx+ey+f=0$) of ellipse with a possible tilt. After the estimation, the tilt is removed from the ellipse (using a rotation matrix), and the rest of the parameters that describe an ellipse are then extracted from the conic representation. Ellipse Fit outputs a structure that defines the best fit. If no ellipse is detected—neither parabola nor hyperbola—an empty structure is returned. Other methods, such as Ellipse Direct Fit [19] and Ellipse Taubin Fit [20], were also tested. By comparison, Ellipse Fit seemed to be simple and straightforward to implement due to its output structure. However, the execution time for Ellipse Fit was slower. The execution times for Ellipse Fit, Ellipse Direct Fit and Ellipse Taubin Fit were approximated to $3.59\times 10^{-4}$, $2.03\times 10^{-4}$ and $1.75\times 10^{-4}$, respectively.

Figure 6b shows the result of the ellipsoidal fitting. The product of the semi-major and semi-minor axes and π gives the area of the ellipse, and thus the surface area of the liquid in pixels. Since we assume the glass is conical, we use the general volumetric equation for cone ($Volume=\left(1/3\right)\cdot \pi \cdot h\cdot (r_{1}^{2}+r_{2}^{2}+\left(r_{1}\cdot r_{2}\right))$, where: *h* is the height between two levels and $r_{1}$ & $r_{2}$ are their respective radii) to calculate the volume between top level and the consequent levels. Value for radius is calculated using $\sqrt{}\left(area\_of\_ellipse/\pi \right)$. The height of the horizontal line tangential to the top of the ellipse is the level in pixels.

### 4.3. Dataset

To infer the relationship between ground truth measurements in millimeters and the derived measurement in pixels, we captured images at various liquid levels in the glass. The images were labeled accordingly and processed as described above. These images are obtainable at [21]. Table 2 shows the sequence of measurements derived from the images. The algorithm could not derive measurements from *image 18*. Outliers were identified and removed before fitting. These errors were a result of bad image quality and edge handling.

**Table 2:** Ground truth, and the derived measurement results. These images are obtainable at [21].

	Ground Truth			Measurement		
Image No.	Level of Water (mm)	Diameter at Level (mm)	Calc. Vol (mL)	Derived Level (Pixels)	Derived Surface Area (Pixels 2)	Derived Volume (mL)
1	60	64	193	146	8215	193
2	60	64	193	11	37173	-
3	60	64	193	147	8019	195
4	50	67	169	133	12106	176
5	50	67	169	132	9932	172
6	50	67	169	133	11596	176
7	40	69	139	109	13895	140
8	40	69	139	110	16630	144
9	40	69	139	111	18662	146
10	30	71	108	74	28001	103
11	30	71	108	75	30119	104
12	30	71	108	76	28314	105
13	20	72	73	43	41572	76
14	20	72	73	44	40306	76
15	20	72	73	44	44878	77
16	10	73	37	64	21279	-
17	10	73	37	80	20109	-
18	10	73	37	-	-	-

Data identified as outliers and exempted from analysis. Could not obtain parameters.

## 5. Results and Discussion

**Figure 7:**
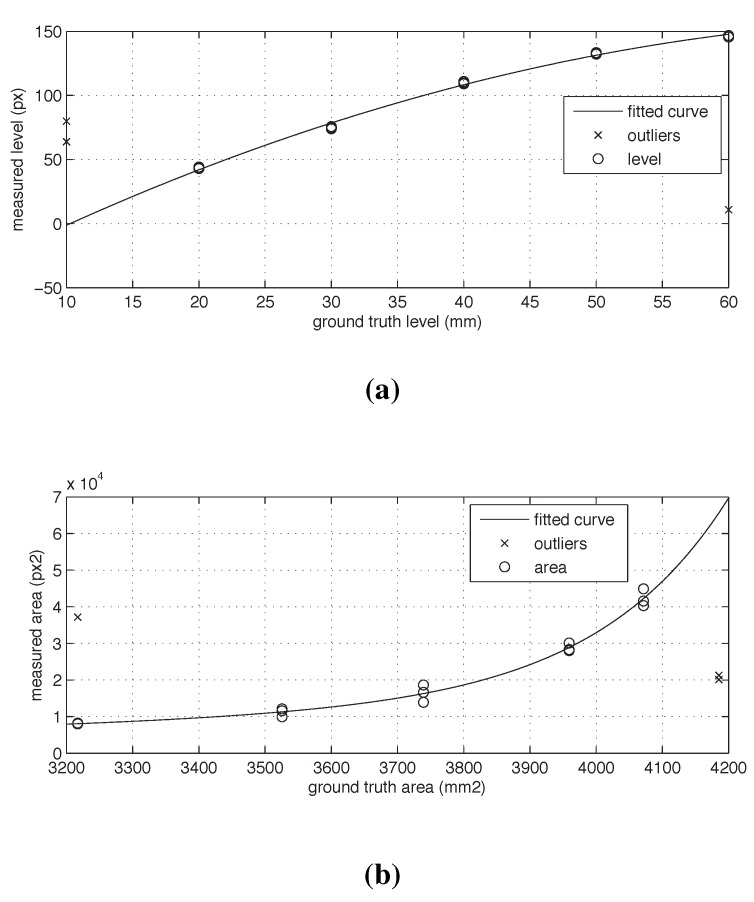
(**a**) Liquid level data plot; (**b**) Liquid surface area data plot.

By plotting the *derived* level against *ground truth*, Figure 7a, we observed a quadratic form with a positive correlation due to the skewed effect of the camera angle and the shape of the glass. A quadratic polynomial fit, *poly2*, was used to derive the linear model in Equation (1) with stdError=1.49 and $R^{2}=0.99.$ Equation (1) was used to infer the *derived* level from pixels to millimeters.

(1) $$DerivedHeightFit\left(x\right)=p_{1}\cdot x^{2}+p_{2}\cdot x+p_{3}$$

where coefficients: $p_{1}=1.68\times 10^{-3},p_{2}=4.71\times 10^{-2},p_{3}=1.54\times 10^{1}$ and *x* is the derived height in pixels.

Given the radius of the glass at the liquid level, we calculated the sectional area of the glass. The result is graphed with the derived area in pixels, which shows a positive correlation. The pattern that the measurements follow suggests the use of an exponential fitting. This pattern is caused by the shape of the glass and by the camera angle. A second-order exponential fitting was used to derive the general model in Equation (2) with stdError=51.23 and $R^{2}=0.98$. Equation (2) can be used to infer the area from *px*${}^{2}$ to *mm*${}^{2}$.

(2) DerivedAreaFit(x)=a·exp(b·x)+c·exp(d·x)

where coefficients: $a=3.67\times 10^{3},b=2.51\times 10^{6},c=-3.65\times 10^{3},d=-2.43\times 10^{-4}$ and *x* is the derived area of the ellipse in pixels.

**Figure 8:**
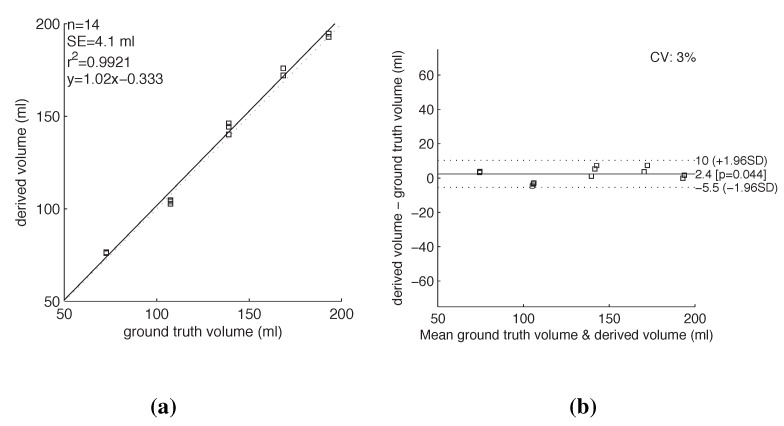
A Bland-Altman plot of the volumetric measurements. This plot was produced in MATLAB using the Bland-Altman function produced by Ran Klein [23].

The actual change in volume for each level was calculated in mL using *Ground truth* measurements by applying the generic equation for cone mentioned in Section 4.2.4. The *derived* measurements in px are converted to standard metrics using Equations (1) and (2) and used to infer the change in volume for each level in ml. To determine whether the two measurement groups (ground truth and inferred measurements) agree closely enough, we computed their differences. Figure 8 is a plot of the two groups and the residuals of the inferred result to ground truth. We get the standard error of mean (SE) of 4.1 mL and a coefficient of variation (CV) of 3%. CV is the standard deviation of the mean values in percentage. With a restriction of 1 L/day, we obtain a 30 mL error per day. In a study amongst patients with hemodiaysis [22], self-reporting showed up to 29% noncompliance in hemodialysis patients, even after intervention. An achievable 3% error in using the proposed method is therefore acceptable.

Digital image processing can be time, memory and CPU intensive. In this work, we used a MATLAB environment running on a 2.5 GHz Intel Core i5 Apple computer to process the images. It took 438 ms on average in executing the image processes required to infer the volume from each image. During execution, the CPU usage increased by 40% (bearing in mind that the program is not optimized for memory usage). It can be a challenge to run the same application on a tiny micro-controller environment. CPU utilization (the amount of time not in the idle task) should be minimum to maximize the device uptime. It should not be necessary to sample the images at a fixed time interval but preferably on event based. Incorporating a motion sensor to track the physical movement of the vessel can help to significantly reduce CPU utilization. It allows that the inferential processes are initiated only when it is useful (*i.e.*, when the device is in a balanced position and the volume content is stable). Further optimization routines can be implemented to keep the sensor module in idle state when the device is not in use. A real-time application is desirable to provide on-spot feedback to the patient that can have impact on their immediate behaviour.

## 6. Options for Future Work

Further experiments should be conducted to evaluate the robustness of the system. The segmentation process can be improved using other morphological methods. Stepwise experimentations can be done with different fluid opacity to evaluate the edge detection methodology. The angle of the camera can be changed in steps in order to check whether the ellipsoidal fittings will provide similar results. Experiments with different glass diameter and with various shapes of the vessel will also prove the robustness of the proposed method. An infrared (IR) camera can be used with an IR source to ensure visibility in poor lighting conditions. The camera device can be miniaturized, integrated and tested.

## 7. Summary and Conclusions

We attempted to infer volumetric measurements using a camera on a conically shaped glass. The results are reasonably correlated to the ground-truth. The inferred method has an acceptable error margin as it depends on the goodness of the fit between the observed measurement and the result of the model. However, we anticipate that the error could vary depending on the cup size. We expect this method to be configurable for different cup sizes. It is possible to miniaturize the camera module, which will make it suitable for both home and ambulatory use. On the other hand, image processes can be CPU intensive; therefore, the hardware specifications should be carefully considered. A motion sensor can be used to monitor the physical movement of the vessel and measure volume only when it is useful. By using the proposed method for inferring liquid volume in a vessel, fluid nonadherenace can be better estimated and data can be used to provide smart behavioral interventions within the context of drinking.
